# Pseudolymphoma of the liver: a case report and literature review

**DOI:** 10.1186/s40792-015-0110-9

**Published:** 2015-10-17

**Authors:** Kazuhiro Taguchi, Shintaro Kuroda, Tsuyoshi Kobayashi, Hirotaka Tashiro, Kohei Ishiyama, Kentaro Ide, Masahiro Ohira, Hiroyuki Tahara, Koji Arihiro, Hideki Ohdan

**Affiliations:** Department of Gastroenterological and Transplant Surgery, Hiroshima University Hospital, 1-2-3 Kasumi Minami ward, Hiroshima, 734-8551 Japan; Department of Anatomical Pathology, Hiroshima University Hospital, 1-2-3 Kasumi Minami ward, Hiroshima, 734-8551 Japan

**Keywords:** Pseudolymphoma, Liver, Laparoscopic hepatectomy

## Abstract

Pseudolymphoma is a benign lymphocytic tumor-like lesion, and its occurrence in the liver is rare. Here, we report the case of a 78-year-old woman with pseudolymphoma of the liver. She had a history of tremors for several years. Therefore, she underwent computed tomography (CT) for screening, and liver tumors were incidentally identified. She did not have any history of liver disease. Liver function test results and tumor marker levels were all within normal limits, and viral markers for hepatitis were negative. Contrast-enhanced CT revealed four nodules measuring up to 13 mm in diameter with ring enhancement in both lobes of the liver. On magnetic resonance imaging, the lesions showed slightly high intensity on T2-weighted images and high intensity on diffusion-weighted images. Because of atypical imaging findings, the tumors could not be definitively diagnosed. Therefore, we performed laparoscopic limited resection of segments 2, 3, 4, and 8 of the liver. The final pathological diagnosis was pseudolymphoma of the liver. The patient has had no signs of recurrence for 6 months after the surgery. Although pseudolymphoma of the liver is rare, it is necessary to consider it in the differential diagnosis of a liver tumor.

## Background

Pseudolymphoma or reactive lymphoid hyperplasia is a benign nonspecific lesion characterized by a marked proliferation of polyclonal lymphocytes forming follicles with an active germinal center [1, 2]. Although pseudolymphoma has been reported in various organs, including the skin [3], lungs [4, 5], eye orbit [6], intestine [7–9], and thyroid [10], its occurrence in the liver is rare. The etiology of pseudolymphoma is yet unknown, and it has been speculated to represent a reactive immunological response to a chronic infection or inflammatory process [11]. In addition, its association with malignancy has been reported [2]. The preoperative diagnosis of pseudolymphoma is difficult because the imaging findings are usually equivocal. Herein, we report the case of a patient with pseudolymphoma of the liver.

## Case presentation

A 78-year-old woman with a history of tremors for several years presented to the Department of Neurology in our hospital. She was admitted to our hospital, and she underwent computed tomography (CT) for screening. The CT scan incidentally revealed multiple lesions in the liver; therefore, a qualitative diagnosis was required.

The results of laboratory examinations, including liver function tests, were all within normal limits. The results were as follows: white blood cell count, 6940/mm^3^; hemoglobin level, 14.1 g/dL; platelet count, 20.8 × 10^4^/mm^3^; serum aspartate aminotransferase level, 21 IU/L; serum alanine aminotransferase level, 19 IU/L; alkaline phosphatase level, 204 IU/L; γ-glutamyl transpeptidase level, 37 IU/L; and total bilirubin level, 1.0 mg/dL. The levels of tumor markers, including alpha-fetoprotein, a protein induced by vitamin K absence, carcinoembryonic antigen, carbohydrate antigen 19-9, and soluble interleukin-2 receptor, were all within normal limits. Viral markers for hepatitis B and C were negative. Levels of immunoglobulin (Ig) G, including IgG4, IgA, and IgM were normal. Tests for antinuclear antibody, anti-DNA antibody, and anti-smooth muscle antibody were negative.

**Fig. 1:**
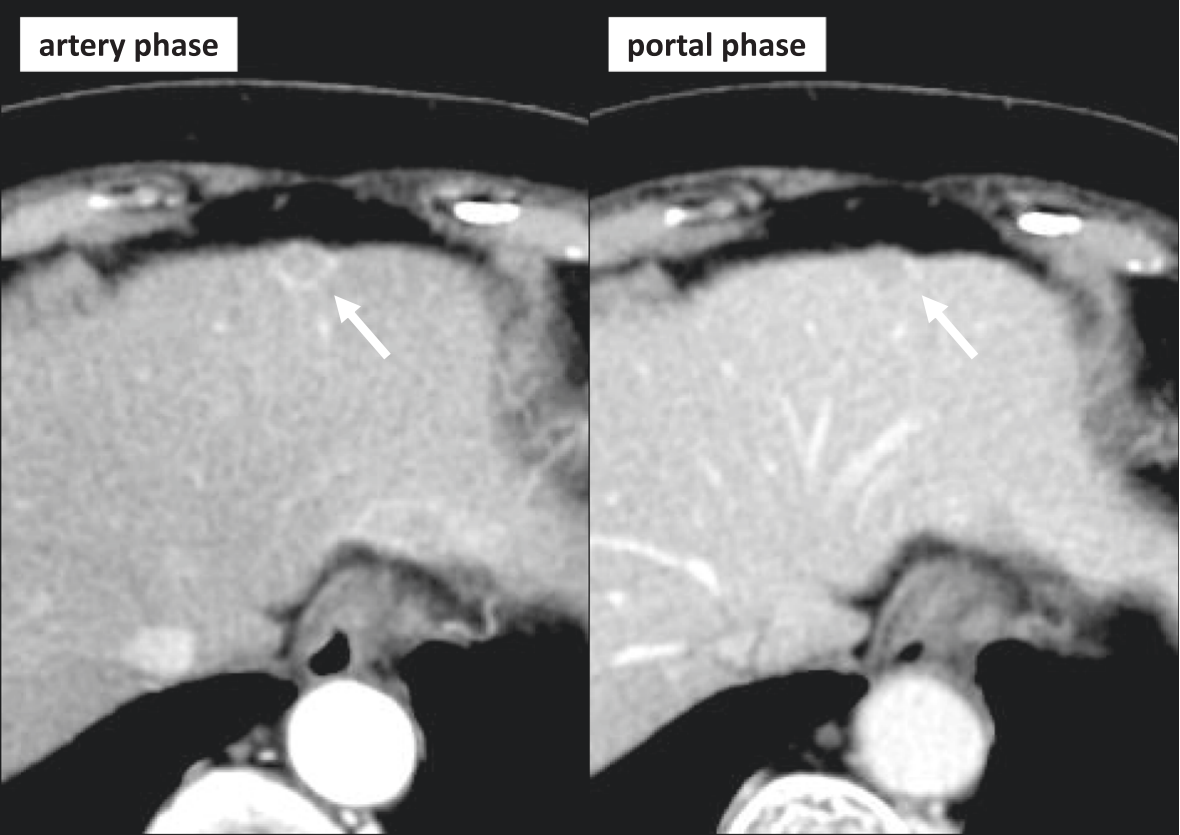
Contrast-enhanced computed tomography showing a nodule with ring enhancement in the arterial phase and subsequent washout in the portal phase in segments 2, 3 (*arrow*), 4, and 8 of the liver

**Fig. 2:**
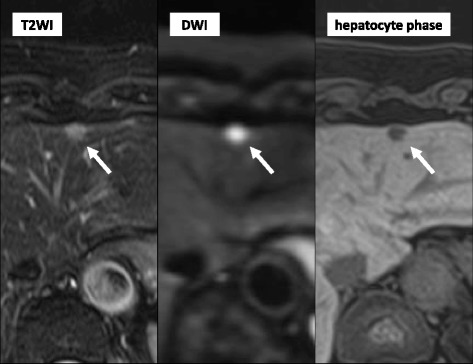
Magnetic resonance imaging (MRI) showing a nodule with slight high intensity on a T2-weighted image and high intensity on a diffusion-weighted image. Gadolinium-ethoxybenzyl-diethylenetriamine pentaacetic acid-enhanced dynamic MRI showing a nodule with defects in the hepatocyte phase in segments 2, 3 (*arrow*), 4, and 8 of the liver

Imaging revealed four nodules measuring up to 13 mm in diameter in segments 2, 3, 4, and 8 of the liver. Ultrasonography revealed low echoic lesions in segments 2 (13 mm in diameter) and 3 (8 mm in diameter) of the liver. On contrast-enhanced ultrasonography using perflubutane, these lesions showed enhancement in the arterial phase, subsequent washout in the portal phase, and defects in the Kupffer phase. On contrast-enhanced CT, these nodules showed ring enhancement in the arterial phase and subsequent washout in the portal phase. In addition, small nodules with the same enhancement pattern were identified in segments 4 and 8 (Fig. 1). CT arterial portography revealed nodular perfusion defects, and CT hepatic arteriography revealed strong enhancement in the early phase and ring enhancement in the late phase. Magnetic resonance imaging (MRI) showed low intensity on T1 weighted images, slightly high intensity on T2 weighted images, and high intensity on diffusion-weighted images. On gadolinium-ethoxybenzyl-diethylenetriamine pentaacetic acid (Gd-EOB-DTPA)-enhanced dynamic MRI, the nodules showed ring enhancement in the arterial phase and defects in the hepatocyte phase (Fig. 2). The patient also underwent whole-body positron emission tomography-computed tomography, and all lesions had standardized uptake values up to 4.6.

Because of atypical imaging findings, these tumors could not be definitively diagnosed. The differential diagnosis included hepatocellular carcinoma (HCC), metastatic liver tumor (unknown primary origin), malignant lymphoma, inflammatory pseudotumor, and pseudolymphoma. We performed laparoscopic limited resection of segments 2, 3, 4, and 8 of the liver.

**Fig. 3:**
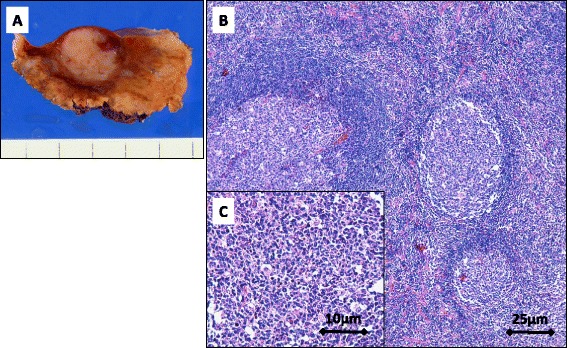
Image of a resected liver specimen showing a well-circumscribed, ash-colored tumor (**a**). On histological analysis, many aggregated lymphoid follicles are seen with germinal centers consisting of lymphocytic or plasmacytic cells without atypia (**b**: *scale bar*, 25 μm, **c**: *scale bar* 10 μm)

**Fig. 4:**
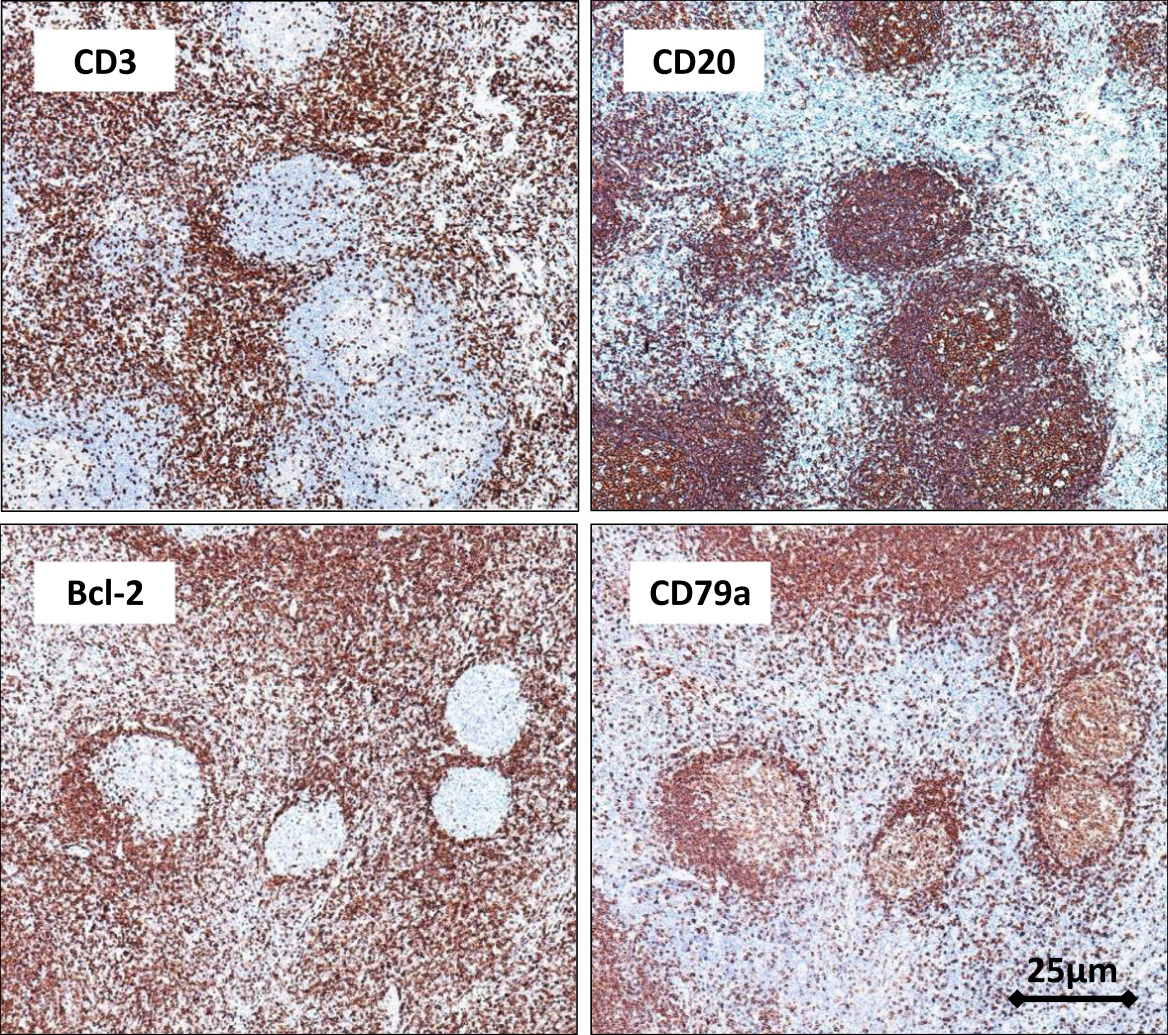
Immunohistochemical analysis of the tumor. CD3-positive cells are mainly localized in the parafollicular area, and CD20 and CD79a immunostaining is positive in B follicles, while Bcl-2 staining is negative (*scale bar*, 25 μm)

On visual examination of the resected liver specimens, the tumors appeared well circumscribed and ash colored (Fig. 3). On histopathological examination of the tumors, many aggregated lymphoid follicles were noted with germinal centers consisting of lymphocytic or plasmacytic cells without atypia along with fibrocollagenous and hyalinized stroma. The lymphoid follicles varied in size and shape, and the germinal centers included small or large lymphoid cells and tingible body macrophages. On immunohistochemical examination of the tumors, CD3-positive cells were mainly localized in the parafollicular area, and CD20 and 79a immunostaining was positive in B follicles, while Bcl-2 staining was negative (Fig. 4). Thus, the possibility of well-defined follicular lymphoma was excluded, and pseudolymphoma of the liver was finally diagnosed. The patient has had no signs of recurrence for 6 months after the surgery.

### Discussion

We reported the case of a patient with pseudolymphoma of the liver. Pseudolymphoma is a nodular lesion considered to result from a reactive immunological response; however, the etiology of pseudolymphoma is yet unknown [1]. It was first described in the lungs by Saltzstein et al. in 1963 as a lymphocytic tumor associated with inflammation and with no evidence of systemic dissemination [12]. Pseudolymphoma of the liver is rare and was first reported by Snover et al. in 1981 [13]. Pseudolymphoma is defined histologically as the aggregation of lymphoid follicles typically with reactive hyperplasia of germinal centers showing proliferation of polyclonal lymphocytes without atypia.

We reviewed the PubMed database from 1985 to 2014, using the keywords “liver,” “pseudolymphoma,” and “lymphoid hyperplasia,” and we found 50 cases of pseudolymphoma of the liver. These cases and our case are summarized in Table 1. The mean age of the patients was 58.9 years (range, 27–85; median, 60). Of the 51 cases, 5 (9.8 %) involved male patients and 46 involved female patients (90.2 %). Among the 51 cases, 14 (27.4 %) had malignant diseases, including gastric cancer [14–17], colon cancer [2, 18, 19], uterine/ovarian cancer [20], renal cell carcinoma [21, 22], pancreatic cancer [17], common bile duct cancer [23], and HCC [24]. Therefore, the pathogenesis of pseudolymphoma of the liver with an associated malignant tumor may be related to an immunological abnormality caused by the malignant tumor itself. However, we cannot confirm the relationship between the presence of a pseudolymphoma of the liver and a malignant tumor. This association may be found frequently on incidental image examinations in patients with malignant diseases.

**Table 1:** Clinical presentation of cases of pseudolymphoma of the liver

	*N* = 51
Age, years	58.9 (27–85)
Sex	
Male	5 (9.8 %)
Female	46 (90.2 %)
Associated cancer	14 (27.4 %)
Gastric cancer	4
Colon cancer	4
Uterine/ovarian cancer	1/1
Renal cell carcinoma	2
Pancreatic cancer	1
Common bile duct cancer	1
HCC	1
Associated autoimmune disease	11 (22.2 %)
Sjogren’s disease	4
Autoimmune thyroiditis	4
Takayasu disease	1
Antiphospholipid syndrome	1
CREST syndrome	1
Associated liver disease	14 (27.5 %)
PBC	6
Chronic viral hepatitis	6
NASH	2
Preoperative diagnosis	41 (described)
HCC	19
Metastatic liver tumor	13
CCC	4
Pseudolymphoma	3
Others	2

Data are presented as mean (range), number (percentage), or number

*HCC* hepatocellular carcinoma, *PBC* primary biliary cirrhosis, *NASH* nonalcoholic steatohepatitis, *CCC* cholangiocellular carcinoma

Because pseudolymphoma is a lymphoid reaction, it has been suggested that immunological dysregulation is associated with pseudolymphoma [24]. Among the 51 cases reviewed, 11 (22.2 %) had extrahepatic autoimmune diseases, including Sjogren’s syndrome in 4 cases [25, 26], autoimmune thyroiditis in 4 cases [24, 27–29], Takayasu aortitis in 1 case [24], antiphospholipid syndrome in 1 case, and CREST syndrome (limited cutaneous form of systemic scleroderma) in 1 case [1]. However, the present patient did not have any autoimmune disease.

Among the 51 cases reviewed, 14 (27.5 %) had chronic liver diseases, including primary biliary cirrhosis in 6 cases [1, 16, 24, 26, 30], viral hepatitis in 6 cases [24, 31, 32], and nonalcoholic steatohepatitis in 2 cases [33, 34]. Pseudolymphoma has been reported to develop after interferon treatment for chronic hepatitis [31, 35]. This finding supports the inflammatory nature of the lesion. Moreover, lymphoid follicles are generally not identified in the portal area of a normal liver but are found in a liver with chronic hepatitis. Therefore, pseudolymphoma of the liver has been reported to be associated with hepatitis [24, 32]. However, the present patient did not have any liver disease, including chronic hepatitis.

Imaging findings of pseudolymphoma resemble those of other vascular tumors of the liver, such as HCC, cholangiocarcinoma, and metastatic liver tumor; therefore, preoperative diagnosis of pseudolymphoma of the liver is extremely difficult with imaging studies alone. In terms of preoperative imaging findings, it has been reported that most pseudolymphomas appear as low echoic lesions with or without well-defined margins on ultrasonography and as lesions having a low density on plain CT [36]. In this case, the lesions showed defects in the Kupffer phase on contrast-enhanced ultrasonography using perflubutane. Perflubutane is phagocytosed by macrophages, including Kupffer cells. A pseudolymphoma is a non-malignant tumor and lymphoid hyperplasia, but pseudolymphoma lesions in the liver replace normal liver tissue. Therefore, these lesions have fewer macrophages, including Kupffer cells, than normal liver tissue, and these lesions may demonstrate a defect in the Kupffer phase. Additionally, the lesions have variable density on contrast CT, and many show enhancement in the early phase and wash out in the late phase [32, 37]. On MRI, most of the lesions show low intensity on T1-weighted images, high intensity on T2-weighted images, hyperintensity on diffusion-weighted images, and hypointensity on T1-weighted images in the hepatocyte phase with Gd-EOB-DTPA enhancement [37]. Pseudolymphoma of the liver may mimic a primary or metastatic hepatic malignancy radiologically, especially in patients with chronic hepatitis or internal malignancies. Among the 51 reviewed cases, 19 had been misdiagnosed with HCC preoperatively. Our patient did not have any risk factors for HCC. Additionally, the patient’s liver was not cirrhotic and the levels of tumor markers were within normal limits. However, CT and MRI revealed multiple tumors with ring enhancement. Therefore, malignancy could not be excluded. We considered hepatic biopsy; however, we believed that it would be insufficient to obtain an accurate diagnosis and there was a possibility of needle implantation of malignant tumor cells. Therefore, we decided to perform hepatectomy without biopsy.

Histologically, pseudolymphoma consists of hyperplastic lymphoid follicles, lymphocytes, and other inflammatory cells, and an accurate diagnosis of pseudolymphoma relies on immunohistochemical analysis [38]. Immunohistochemical staining shows positive results for CD3, CD4, and CD8 (T cell markers) and CD20 and CD79a (B cell markers), indicating the polyclonality of the tumor. Additionally, CD20 positive B cells are predominantly located within the lymphoid follicles, and CD3 positive T cells are predominantly located in the peri and interfollicular areas. However, the lymphocytes within the germinal centers are negative for Bcl-2, indicating the reactive and nonneoplastic nature of the tumor [20].

The difference between pseudolymphoma and malignant lymphoma is significant. Although the precise etiology and pathogenesis of pseudolymphoma remain unknown, its prognosis is much better than that of malignant lymphoma, especially follicular lymphoma. Follicular lymphoma includes atypical cells and shows monoclonal proliferation. Additionally, Bcl-2 expression in lymphoid follicles is distinct for follicular lymphoma. Moreover, in situ hybridization demonstrates intermixed kappa and lambda light chains within interfollicular plasma cells. DNA analysis helps to distinguish these lesions from those capable of malignant clonal lymphoproliferation by ruling out monoclonal rearrangements of immunoglobulin heavy chains or T cell receptor beta and gamma genes [32].

## Conclusions

In conclusion, pseudolymphoma of the liver should be considered in the differential diagnosis of small hepatic tumors, especially in female patients and patients with no risk factors for hepatocellular carcinoma. Because it is difficult to preoperatively distinguish pseudolymphoma from malignant tumors and metastases on the basis of imaging findings, careful follow-up of patients is essential and surgical intervention should be the choice of treatment on suspicion of malignancy.

## Consent

Consent was obtained from the patient for the publication of this case report.

## Abbreviations

CT: computed tomography
Gd-EOB-DTPA: gadolinium-ethoxybenzyl-diethylenetriamine pentaacetic acid
HCC: hepatocellular carcinoma
MRI: magnetic resonance imaging
